# Evaluation of the Epidemic Situation of Anthrax in Armenia Over the Last Decade

**DOI:** 10.1111/zph.13181

**Published:** 2024-09-09

**Authors:** Satenik Kharatyan, Khachik Sargsyan, Hasmik Elbakyan, Varduhi Hakobyan, Vazgen Sargsyan, Gayane Chobanyan, Manvel Badalyan, Tigran Markosyan

**Affiliations:** ^1^ Scientific Center for Risks Assessment and Analysis in Food Safety Area of the Ministry of Economy of the Republic of Armenia Yerevan Republic of Armenia; ^2^ Armenian National Agrarian University Yerevan Republic of Armenia

**Keywords:** animals, anthrax, one health, vaccination, zoonoses

## Abstract

**Introduction:**

Anthrax is a World Organisation for Animal Health (WOAH)‐listed disease that must be reported upon confirmation based on the Terrestrial Animal Health Code. Anthrax poses a serious health issue for unvaccinated livestock, is a threat to humans through interaction with contaminated livestock and animal products and is endemic in many areas throughout the world, including the Transcaucasian Region. Despite several control and eradication efforts that have been implemented by the government of the Republic of Armenia (RA), sporadic cases of anthrax are still reported. We sought to understand the epidemic situation of anthrax in RA during the last 10 years (2012–2023) based on analysis of outbreaks and reported cases in cattle and humans.

**Methods:**

We collected and evaluated officially reported data from human and animal cases, such as time, location, animal species, disease intensity and spread radius. The data and various parameters were mapped using ArcGIS to prepare a viable risk assessment.

**Results:**

Based on the officially available data and reports, there have been 80 human cases and 55 animal cases of anthrax confirmed in RA from 2012 to 2023. We also identified the presence of anthrax spores in soil and environmental samples near animal burial sites in RA in 2015–2017 through polymerase chain reaction (PCR) testing. Upon comparing the human and animal cases by frequency and intensity, the human cases are directly proportional to the animal husbandry practices performed in RA.

**Conclusion:**

The detection of the anthrax pathogen at the burial sites highlights the continued threat in these areas. Thus, it is imperative to secure and monitor any areas that have been used for anthrax burial and limit the movement of animals in these areas. In the future, legislation should be updated to prioritise incineration of anthrax‐infected carcasses instead of burial to limit further exposure to animals and humans.

**Summary**

- This retrospective study identified the continued threat of anthrax to humans and animals with a close temporal correlation through the interaction between humans and animals.
- Observed seasonal anthrax cases indicate that climate‐related factors (precipitation and ambient temperature) may play a critical role in the occurrence of outbreaks, with variations among locations and suggest that preventative vaccinations should be carried out in animals prior to their movement to pastures near the end of spring and the beginning of summer.
- Carcass disposal (burial or incineration) is a significant issue that has a high socio‐economic burden for veterinary services and ecologists and should play a key role in control and eradication of anthrax.

## 1 Introduction

Anthrax is a serious zoonotic disease that affects mammals and, rarely, birds. It is caused by a spore‐forming bacterium, *Bacillus anthracis*, which animals usually acquire from contaminated vegetation, soil or feed products such as bone meal. Anthrax spores are extremely resistant to inactivation and can survive in the environment for decades. Species of animals differ in their susceptibility to anthrax. Domesticated and wild herbivores tend to be extremely susceptible and often die rapidly, while omnivores and carnivores are more resistant to developing clinical signs and may recover without treatment if they become ill (Rovid Spickler 2017). Each animal responds differently on their progression from infection to septicaemia, with death occurring when the amount of the circulating bacteria reaches a high enough level in the blood that correlates with the amount of toxin present (Lincoln et al. 1967).

Anthrax spores are usually ingested, inhaled or enter the body through skin abrasions or cuts, after which they can germinate, multiply and produce toxin. Insects can transmit the bacterium between animals, but transmission does not typically spread from animal to animal or human to human (Beyer and Turnbull 2009; Carlson et al. 2018; Fasanella et al. 2013; Finke et al. 2020; Hugh‐Jones and Blackburn 2009; WHO 2008). Humans most often acquire the disease through handling of sick animals or through the consumption of contaminated meat (WHO 2008).

Immediately prior to death, animals may show signs of high fever and blood may be present around the nose, mouth and anus of carcasses; although these signs may be absent and should not be relied on to diagnose anthrax. If livestock die suddenly, even when there is no history of anthrax in the area, anthrax should be considered and included in diagnostic tests to determine the cause.

To prevent the spread of anthrax, it is important that all cattle and sheep that die unexpectedly without obvious clinical signs are tested for anthrax before they are transported, processed, or buried. This testing will reduce the risk of human exposure and minimise further contamination of the environment if anthrax is confirmed (Beyer and Turnbull 2009).

Anthrax incidence in each locality is related to temperature, precipitation or drought, soil, vegetation, host condition and population density (Fasanella et al. 2013; Hugh‐Jones and Blackburn 2009). Anthrax spores may remain dormant in the soil for long periods and resurface when the soil is disturbed, such as by flooding, torrential rains or landslides. The local weather condition of an area may directly or indirectly influence the possibilities for animals to be exposed to *B. anthracis* spores (e.g., grazing closer to the soil in dry periods when grasses are short or sparse (Carlson et al. 2018; Finke et al. 2020; Mwakapeje et al. 2018)). Anthrax cases usually occur between April and October, when the animals have not developed full immunity following vaccination, which starts between January and June. This vaccination schedule allows animals to graze in areas with a high density of susceptible animals, in or near high‐risk areas including cattle cemeteries and slaughterhouses, without full immunity.

Anthrax is listed in the Class B of infectious diseases ‘with the high possibility of transmission and fatality in the article list of quarantine of especially dangerous and notifiable diseases of animals’ under the Republic of Armenia law on Veterinary Medicine ‘HO‐137‐N’ dated 21 June 2014 (Ministry of Agriculture 2014). Under this policy, certain infectious diseases, including anthrax, must be reported to the national veterinary services within 24 h of initial disease confirmation and require compulsory notification to the World Organisation for Animal Health (WOAH).

Anthrax is present in most parts of the world, but the frequency of outbreaks varies. Globally, sustained anthrax is reported across parts of North America, West and Sub‐Saharan Africa, Central Asia, China and Southeast Asia (Carlson et al. 2019). In the European Union/European Economic Area, there were only 19 confirmed anthrax cases reported to the European Surveillance System between 2016 and 2020, ranging from one to six cases per year (Control 2020).

Anthrax is also an issue in the Transcaucasian Region, which includes the Republic of Armenia (further referred to as Armenia) and neighbouring countries of Azerbaijan, Georgia, Türkiye and Iran (Amiri et al. 2021; Armenia 2023; Australia 2020; Fasanella et al. 2010; Haykuni 2016; Hugh‐Jones and Blackburn 2009). However, it is thought that the full extent of livestock cases of anthrax is significantly underreported. Based on the reports of anthrax in the World Animal Health Information System (WAHIS) of WOAH, Georgia, Iran and Türkiye have continually reported anthrax cases yearly since 2005, with Azerbaijan reporting cases in 2006–2008, 2012–2013 and 2019–2023 (WOAH 2023).

In Armenia, most animals are kept on household farms, where animal husbandry practices and feeding are based on traditional knowledge. Armenia's low‐lying areas are popular year‐round pastures, while in the foothills and mountainous areas, animals are usually confined in stables and/or graze on local or summer pastures. Local animal grazing generally occurs within the vicinity of the village, with animals returning each day to the farm for milking. Alternative grazing methods include moving animals to a specific area, usually remote mountain pastures for a period of time before returning home. The climate in Armenia is continental, with cold winters and hot summers, and precipitation is usually not abundant.

Backyard farms play a crucial role in rural households by providing essential quantities of food both for home consumption, as well as for sale or trade in the local market (milk, meat and eggs) (Agriculture 2013). Approximately 80% of Armenian household farms maintain 1–3 cattle, 3–4 pigs, 3–10 sheep and 15 poultry. The Yezidis, a national minority population in Armenia run small household farms with large herds of sheep (up to 1000). There are also medium‐sized commercial farms that house approximately 50 or more cattle, 60–90 pigs or 50–150 sheep and large commercial farms that can house up to 1500 pigs or 1000 sheep.

In endemic regions, anthrax can be controlled with vaccination of susceptible animals. In Armenia, cattle, sheep and horses are vaccinated annually with a Russian‐manufactured anthrax vaccine (strain 55) for livestock. During 2022, a total of 549,152 cattle were vaccinated in the spring, followed by a re‐vaccination of 364,144 cattle in high‐risk areas, which accounts for 68% of all cattle in Armenia. Additionally, 10,685 horses and 38,092 sheep from the high‐risk areas (Gegharkunik and Shirak Regions) were vaccinated, which accounts for only 6% of all sheep and 79% of horses in Armenia. Even with vaccination in high‐risk areas of Armenia, sporadic cases of anthrax are still reported in those areas, and surveys are only performed following the report of an outbreak. The last anthrax outbreak in Armenia occurred between 22 June and 29 June 2021 in the Gegharkunik Region.

In this paper, we evaluate the status of anthrax in Armenia from 2012 to 2023 using historical data and reports from both human and animal health sectors with the following objectives: (1) describe the spatiotemporal patterns and epidemiological characteristics of anthrax in humans and livestock across the regions; (2) analyse vaccine coverage in livestock to anthrax reports in human and livestock cases and (3) map the high‐risk area according to densities of humans and livestock, coverage of vaccination and areas with the historical animal burial locations.

## 2 Materials and Methods

### 2.1 Study Design and Ethical Approval

The study design was approved in advance by the Scientific Council of the Scientific Center for Risks Assessment and Analysis in Food Safety Area of the Ministry of Economy of the Republic of Armenia (March 15, 2022; Protocol No. 3) and work was performed in accordance with the relevant institutional and national guidelines set forth by the government of the Republic of Armenia. Human case data and outbreak information were provided by the National Centers for Disease Control and Prevention (NCDCP), Ministry of Health (MoH) of Armenia.

In Armenia, within the framework of the Agricultural Animal Vaccination programme, cattle, horses and sheep are vaccinated against anthrax once a year with the 55‐strain anti‐anthrax vaccine in designated high‐risk areas, and there is no regular surveillance of anthrax. Testing is only performed upon notification of a suspected case of disease.

### 2.2 Laboratory Diagnostics

All reports regarding anthrax cases were collected from the NCDCP of the MoH and the Reference Laboratory of Especially Dangerous Pathogens (RLEDP) of the Food Safety Inspection Body (FSIB).

According to RA MoH of 2013 and according to the decision N 2750‐A of October 16 ‘On the approval of standard definitions of infectious diseases, food poisoning cases’, the presence of the following indicators is necessary for the diagnosis of anthrax:

- Suspected: A case that meets the clinical criteria is epidemiologically linked to a confirmed or suspected diseased animal or to the consumption of contaminated food from those animals.
- Probable—suspected case with a positive skin allergy test among unvaccinated persons.
- Confirmed: a suspected case confirmed by laboratory testing, such as microscopic, microbiological, biological, serological and PCR methods (Health 2013).

NCDCP can perform microbiological, serological and molecular genetic methods to properly diagnose anthrax in humans. Sampling is performed based on the clinical form of the disease, which can include collecting swabs from open wounds, blood, respiratory secretions, pleural or ascitic fluid, faeces and cerebrospinal fluid. Most clinical laboratories can only provide a preliminary result, but definitive diagnosis for humans requires testing by polymerase chain reaction (PCR) on wound, blood or serum samples and an immunohistological examination performed at the Laboratory of Especially Dangerous Infections and Live Cultures at NCDCP (Health 2019).

When animals in the regions are presenting with symptoms of weakness and fever, community veterinarians and the FSIB inspectors observe the animals and sample pathological material from an animal that has been slaughtered or forcibly skinned and send it to RLEDP for diagnosis, observing all safety measures, including use of proper personal protection equipment and following predefined protocols for sample collection and delivery (Agriculture 2013).

Confirmation is performed by Gram stain and Ascoli precipitation testing as recommended by WOAH (WHO 2008; WOAH 2018) and PCR testing using Biofire Defense Anthrax PCR Kit Target 1, 2, 3, to amplify the pathogenic plasmids pX01 and pX02, per manufacturer's directions (Ellerbrok et al. 2002).

Field samples from soil and other environmental objects were collected during outbreaks and from burial grounds for testing from five communities (Orgoy, Vardenis, Sevan, Spitak, and Akhuryan) for a total of 60 samples collected. DNA was isolated using the Soil DNA Isolation Kit (Norgen Biotek Corp., Canada) and the extracted DNA was amplified by PCR using the Biofire Defence Anthrax PCR kit detection as previously described (Haykuni 2016).

### 2.3 Data Collection

To understand the epidemic situation of anthrax in Armenia during the last 10 years (2012–2021), we retrospectively evaluated annual reports, registers and data collected during two community missions in each of five different regions based on the confirmation of historical cases in these regions. The five regions included Armavir, Shirak, Lori, Kotayk and Gegharkunik. The information collected from the official sources of FSIB included: species, sick or death with dates, vaccination status and location. During the community missions, meetings were organised with the regional veterinarians and farmers to clarify how the control measures would be carried out and how the process of sharing information between farmers, veterinarians and NCDCP experts was expected to be accomplished. Each of these individuals was provided with a summary of the activities to be performed during emergency situations and outbreaks.

Human case data were collected from the NCDCP of MoH of Armenia and included the number of cases, regions and the month/year that the epidemiology information was collected, all compiled into Microsoft Excel for analysis.

Human and animal cases were compared with time, location, animal species, disease intensity and spread radius, after which all these data were mapped by ArcGIS to perform a risk assessment. Data were analysed to correlate several factors including animal and human prevalence, animal movement control, presence of animal slaughterhouses, buried animal's approximate locations, vaccination coverage and other potential risks.

### 2.4 Study Population and Area

**FIGURE 1:**
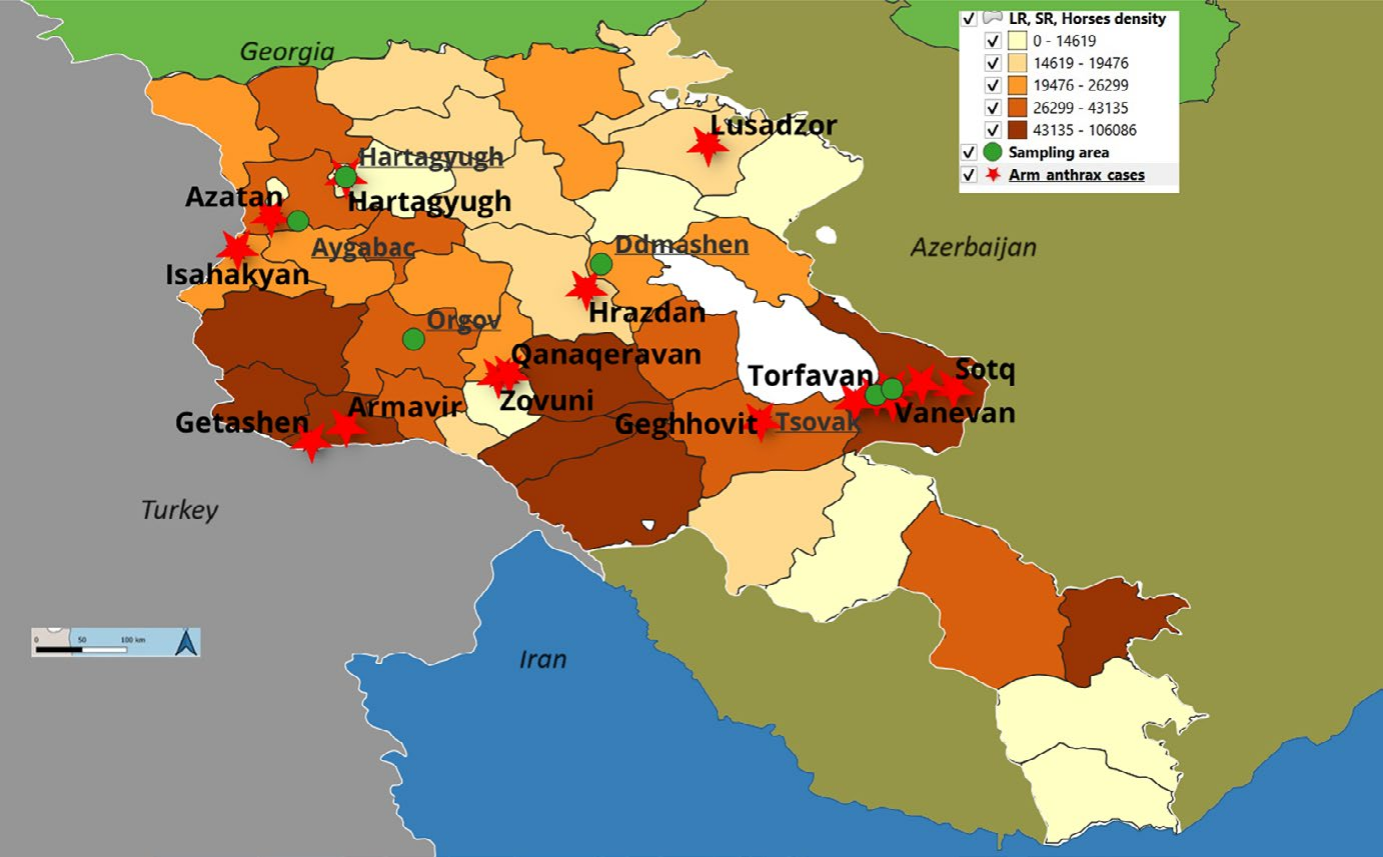
Anthrax cases in animals and humans from 2012 to 2021, with sampling from high‐risk areas and assessment of anthrax with the susceptible animal's density in Armenia.

Armenia occupies an area of 29,800 km^2^ and is located at the convergence of the Southern Caucasus and Asia Minor, in the northeastern portion of the Armenian Plateau (Figure 1). Armenia borders Georgia to the north, Azerbaijan to the east, Iran to the south and Turkey to the west. The average altitude is 1800 m above sea level. The total human population in Armenia is approximately 3 million, with the national animal census reporting 491,370 cattle, 680,843 sheep and goats (small ruminants [SR]) and 14,740 horses in 2023. The individual numbers of humans and animals in the five anthrax‐affected regions are shown in Table 1.

**TABLE 1:** Meteorological and population census of humans, cattle, horses and small ruminant (SR) by individual regions affected by anthrax (Armenia 2023).

Region	Climate	Annual precipitation levels	Human population (2023)	Animal population (2023)		
				Cattle	SR	Horses
Armavir	Dry continental hot summers and mildly cold winters	Around 305 mm	265,770	53,715	137,271	113
Gegharkunik	Cold and snowy in winter, while the summer is characterised by a warm and humid climate	Between 500 and 600 mm at below 2000 m, while it may reach up to 1000 mm in the mountainous areas	235,075	78,847	95,926	1603
Kotayk	Quite diversified; ranges between arid and semi‐arid climate in the south, and snowy climate in the centre of the region and the north	Less than 200 mm in the dry areas; 400 mm at the peaks in the centre of the region, and 900 mm in the north of the region	282,100	44,718	8515	846
Lori	Extremely cold snowy winters and mild summers	Between 600 and 700 mm	235,537	68,253	9249	2416
Shirak	Extremely cold, snowy winters and mild summers	Reaching up to 700 mm	251,941	65,848	7982	253

Armenia is a mountainous country, and only a small portion of its territory is located below 1000 m above sea level, with no plains. With increasing altitude, the temperatures decrease and precipitation increases, with high snowfall in the winter. At lower elevations, the rains are scarce and the summer is extremely hot. In winter, cold air masses predominate, enter the country from the north, stagnating in the valleys and causing severe frosts. Spring is an unstable season and experiences the most frequent rains overall, while remaining generally arid at lower elevations. Summer is dry and sunny, with afternoon thunderstorms in the mountains.

## 3 Results

**FIGURE 2:**
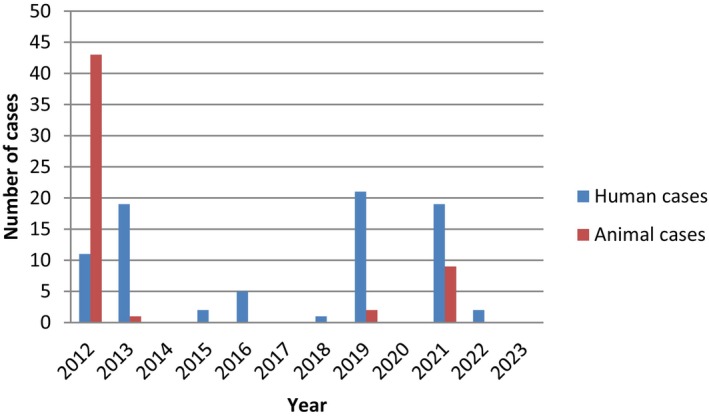
Number of recorded cases of anthrax in humans and animals 2012–2023.

**FIGURE 3:**
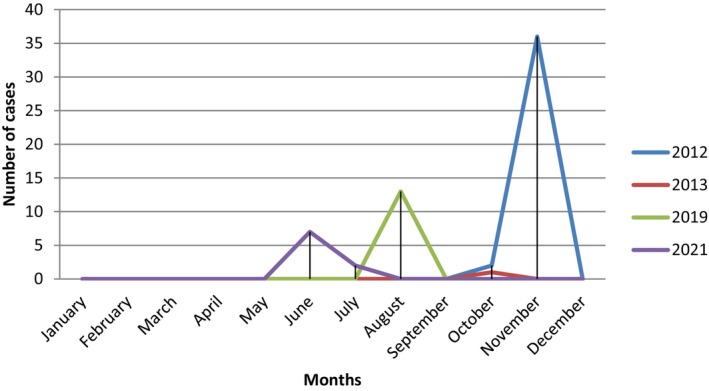
Human and animal anthrax cases by year and month of outbreaks.

According to WOAH annual reports, NCDCP and RLEDP official reports that there have been sporadic outbreaks of anthrax in which humans have also been infected. Over the last decade, there have been 80 confirmed human and 55 confirmed animal cases of anthrax in 2012–2023; 2012 in the Gegharkunik (35 cases), Kotayk (3 cases), Shirak (1 case) and Tavush (2 cases) Regions, 2013 in the Lori Region (Hartagiugh Village, 1 case), 2019 in the Armavir (Getashen, 1 case) and Gegharkunik (Geghahovit, 1 case) Regions and 2021 in the Shirak (Isahakyan, 2 cases) and Gegharkunik (Vanevan, 7 cases, Torfavan, 1 case) Regions. According to available data, all anthrax animals were buried in various livestock cemeteries. Currently, these cemeteries do not have any oversight with biosafety or biosecurity, and the process of mapping them is underway to provide oversight into the future. We found that the reported human anthrax incidence risk over the 2012–2023 per 10,000 populations was much higher in the Gegharkunik Region at 15․4 followed by the Shirak Region at 6․2. All human cases were cutaneous. For susceptible animals, the Gegharkunik Region is also a high‐risk area for anthrax, with the incident risk per 1000 population of cattle was 35.9 followed by the Armavir Region with 20.9 in 2012–2023 (Table 2). Most human cases were registered in 2019, with the majority of anthrax cases in animals in 2012 (Figure 2) followed by no animal cases again until 2019 and 2021 with outbreaks peaking in the summer and fall (Figure 3). Theoretically, unreported cases of other animals could go unreported or undetected since regular surveillance does not presently occur in Armenia. We have recently employed an identification system of cattle, so we can better survey and trace the movement of animals. These data were collected from confirmed outbreaks only and may not represent all true data, so unfortunately our analysis was limited. PCR results on the environmental samples identified *B. anthracis* in four communities across three regions, Vardenis and Sevan of the Gegharkunik Region, Spitak in the Lori Region and Akhuryan in the Shirak Region (Haykuni 2016), which highlights the continued threat in these areas.

**TABLE 2:** Spatial distribution of reported anthrax cases in 2012–2023, based on the human and livestock (cattle, small ruminant [SR], horses) data in the National Information System of Armenia (Armenia 2023).

Region	Estimated human population (2012–2021)	Estimated animal population (2012–2021)			Human cases (2012–2023)	Livestock deaths (2012–2023)		Human (incidence risk per 1000 people)	Animal (incidence risk per 1000 people)	
		Cattle	SR	Horses		Cattle	SR		Cattle	SR
Shirak	241,170	94,496	77,527	376	15	2	1	6.2	2.1	1.3
Gegharkunik	208,510	103,102	96,568	1406	32	37	2	15․4	35.9	2.1
Armavir	265,800	52,770	100,451	121	10	11	0	3.8	20.9	0.0
Kotayk	253,190	55,275	41,960	560	3	3	0	1.2	5.4	0.0
Tavush	124,890	33,848	12,768	1513	0	1	0	0.0	2.9	0.0
Lori	223,400	74,572	28,204	2505	13	1	0	5․8	1.3	0.0
Syunik	138,950	52,589	97,087	2935	8	0	0	5.8	0.0	0.0
Ararat	258,800	37,715	107,621	1188	0	0	0	0.0	0.0	0.0
Vayots Dzor	50,700	16,400	5340	777	0	0	0	0.0	0.0	0.0
Aragatsotn	128,960	56,854	9507	414	0	0	0	0.0	0.0	0.0
Yerevan	1,097,800	3410	4100	86	0	0	0	0.0	0.0	0.0
Total	2,992,170	609,277	648,306	11,023	80	55	3	2.7	9.0	0.5

Analysis of anthrax cases in both humans and animals highlighted the multiple causes and concerns for the common routes of transmission between them. During the outbreak in 2012, FSIB received an alert from the MoH that individuals were suspected of having cutaneous forms of anthrax, which prompted an investigation by the specialists of the Regional Health Service, who determined that prior to detection of the human cases, forced slaughter of cattle had been carried out in violation of the requirement to notify veterinary services. Similarly, on October 4, 2013, it was discovered that the cases of anthrax among seven residents of the Hartagyugh community in the Lori Region were directly related to the slaughter of four animals on 23–24 September 2013. In both cases, the human cases were traced to the animal source of infection, which led to the confiscation of infected meat and meat products from the residents of the community and the neighbouring Shirak Region.

During the outbreak in 2021, there were nine human cases in the Gegharkunik Region and 10 human cases in the Shirak Region, who were all the owners of anthrax‐infected animals and most had been engaged in fur and meat processing of their sick animals.

Following each outbreak of anthrax, the regional veterinarians of FSIB and the epidemiologists of the MoH developed and implemented preventive measures aimed at the elimination of disease. When necessary, a quarantine was announced to isolate the infected farms and communities from uninfected, susceptible animals according to the legal requirements. The movement of animals, as well as the procurement and export of animal husbandry raw materials, were temporarily prohibited at the point of registration of the disease. Relatively susceptible animals were revaccinated and borderline disinfection was carried out in farms and adjacent areas of the infected animals. In all insecure areas, quarantine or partial restrictions were lifted 15 days after final disinfection.

**FIGURE 4:**
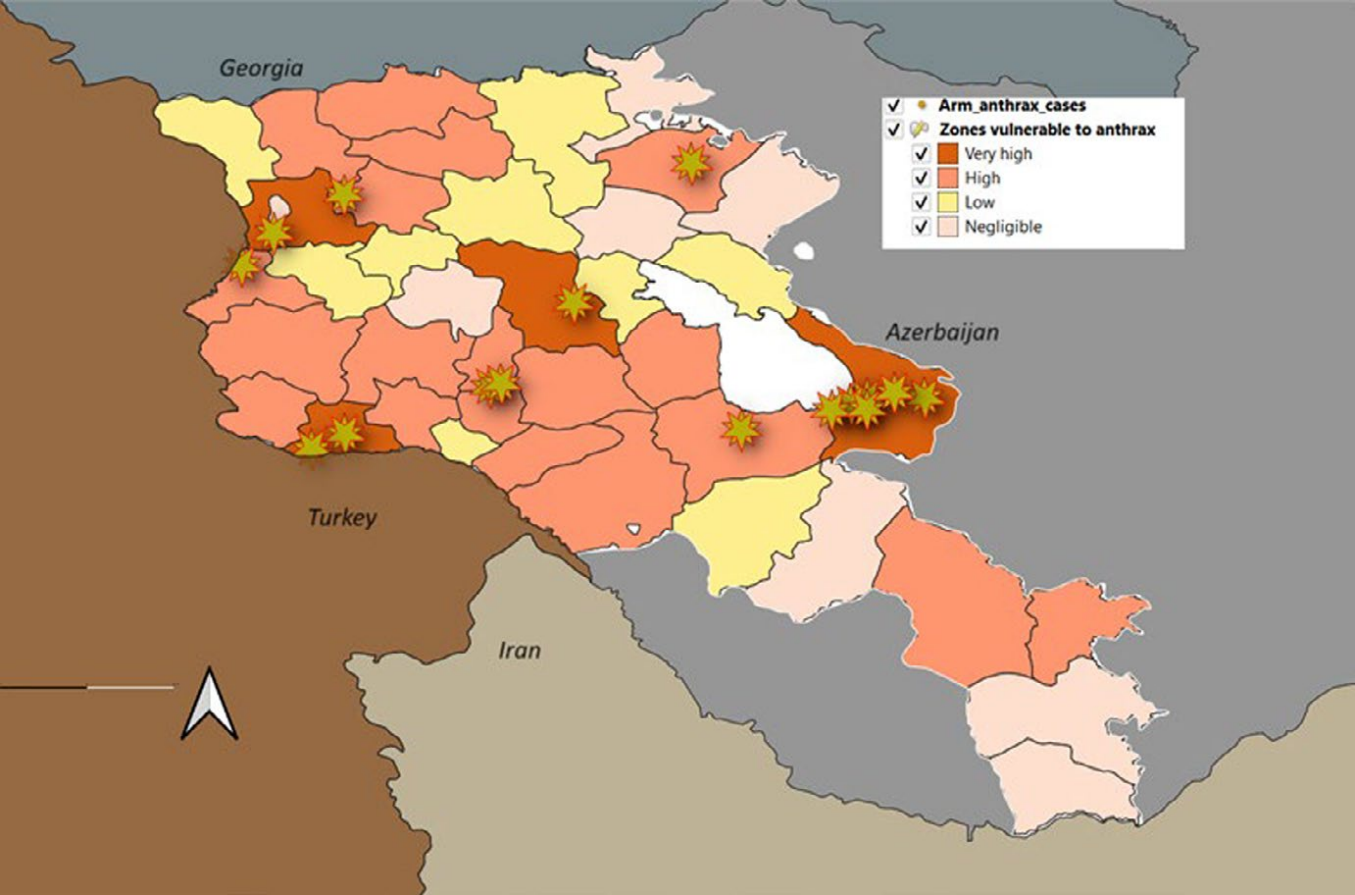
Risk map for anthrax in Armenia.

Based on the available data regarding both human and animal cases, livestock and human densities, husbandry practices and other available epidemiological data, a risk map for Armenia was developed to indicate large areas vulnerable to anthrax outbreak (Figure 4). This figure shows that a significant amount of work remains to be done to protect both the human and animal populations.

## 4 Discussion

This retrospective study shows the ongoing threat and high‐risk concern for anthrax outbreak in Armenia, with human and animal cases showing a close temporal correlation, which is most likely attributed to the ongoing interaction between humans and animals such as types of animal husbandry or occupational exposure. The analysis identified that all anthrax‐positive human cases were butchers, who had recently purchased meats from farmers or the animal slaughterers, and the farmers and animal slaughterers themselves in regions where animal cases with anthrax occurred. The One Health approach to collaboration between MoH and FSIB is extremely important and includes each organisation informing another when a case is confirmed, although this process could be improved with immediate collaboration at the time point of suspicion rather than confirmation. The earlier response by both animal and human health organisations working together in the field could reduce the number of people in contact with sick animals or their contaminated by‐products and allow identification of additional patients and animals to shorten time to prognosis. Animal cases are often unreported because there are no compensation mechanisms for farmers losing an animal to disease, and there is limited education on transmission of disease and on proper personal protective equipment for slaughter and disposal of infected animals. Educational awareness of anthrax and control measures in the field should be implemented with all participants that could be affected by the disease, and compensation for the loss of animals would greatly enhance complying with necessary rules and notification measures.

We also found that anthrax incidence in Armenia in animals and humans primarily occurs in the fall, starting in October, when there are higher amounts of rainfall and in the summer months that are hot and dry, which may be a contributing factor for anthrax outbreaks and transmission in animals (Australia 2020; Carlson et al. 2018). Observed seasonal anthrax cases indicate that climate‐related factors (precipitation and ambient temperature) play a critical role in the occurrence of outbreaks, although there is variation among locations (Amiri et al. 2021; Fasanella et al. 2010; Finke et al. 2020; Shaheenur Islam et al. 2018; WHO 2016).

In order to assess the degree of risk, it is important to note that we identified the disease from noticeably sick and dead animals that were tested and confirmed in reported outbreaks, but there are cases that are unnoticed or unreported without regular surveillance that can contaminate the environment and perpetuate the disease. As human cases are a result of secondary infection following contact with infected animals, there is a disconnect between humans, who are more likely to visit a doctor even with minor skin damage in the nidus of the disease, which can lead to misdiagnosis, and no connection with or tracing back to diseased animals.

Based on our implementation of planned preventive and control measures against anthrax, the epidemic intensity has been reduced to endemic and sporadic manifestations, but more work is needed.

The results from this study emphasise the need for preparedness and development of a strategic plan to combat anthrax. This plan should include preventive vaccination in animals in Armenia prior to their movement to pastures near the end of spring or the beginning of summer. When analysing the most recent cases of anthrax, there was a suggestion that the anthrax vaccinations under the programme in the spring of 2021 were incomplete, which is most likely due to COVID‐related restrictions in Armenia and the adaptation of veterinary services in 2020 due to the new pandemic situation. There have been no reported outbreaks since 2021 and mass vaccinations were performed in 2021 and 2022. Even though there have been no confirmed cases, regular surveillance for anthrax in cattle, small ruminants and horses, is not performed in Armenia and cases may still have occurred without notification.

In addition to the control and eradication of anthrax, carcass disposal (burial or incineration) is a significant issue that has a high socio‐economic burden for veterinary services and ecologists (Carlson et al. 2018; Fasanella et al. 2010). For many years, animals infected with anthrax have been buried without traceability or follow‐up response actions and plans. For example, proper disposal standards for the safe disposal of dead animals include burying in a pit at least 6 ft deep followed by the addition of a 10% formalin disinfectant poured on top of the carcasses (Agriculture 2013; Australia 2020; Tretyakova and Fedotov 1989; WOAH 2023). Further decontamination of the area where a carcass is found and removal of bloody soil should be performed for every case. Areas, where anthrax carcasses are buried, should be readily identifiable with signs and fencing, and the ground should be tested regularly for detection of the pathogen. The epidemiological situation of animals in vulnerable areas contaminated with anthrax when tested by classical microbiological methods is almost impossible, time‐consuming and can be inaccurate. On the contrary, the PCR method has advantages and is considered highly accurate. The use of this method made it possible to detect the spore of the causative agent of the disease, and differences between the virulent and nonvirulent strains in places where it was impossible to detect by microbiological examination. Indeed, the positive detection of environmental sources contaminated with *B. anthracis* identified in five communities reiterates the need for biosafety controls and ongoing monitoring (Haykuni 2016). It should be clarified that while in this case, PCR helped detect positive spores, a negative result does not guarantee a site is safe from disease. The free movement of animals throughout Armenia without restrictions is a risk factor that needs to be addressed to further protect the health of animals and humans.

In addition to the control and eradication of anthrax, in recent times, the use of broad‐spectrum antibiotics as well as disinfectants, such as serotherapy and chemotherapy agents used in veterinary medicine, have led to changes in the biological and clinical properties of the causative agent of the anthrax. In fact, we have noticed a similar pattern following several years of observations, and we included the idea by taking the analysis of the results of other researchers (Dassanayake, Khoo, and An 2021; Hart and Beeching 2001). In animals, anthrax can be accompanied by atypical clinical signs, which is a challenge for veterinarians and makes it difficult to properly diagnose.

We also suggest that the diagnostic capacity in both the public health and veterinary sectors needs improvement with updated modern molecular techniques, which can increase the sensitivity and specificity of testing methods and reduces the need for isolation of pathogens in Armenia. Molecular techniques are rapid and safe to perform in the laboratories and should be added to regular testing mechanisms in Armenia before and after movement of animals and in high‐risk grazing areas to help prevent the spread of disease in animals and the transmission to humans.

## Ethics Statement

The study design was approved in advance by the Scientific Council of the Scientific Center for Risks Assessment and Analysis in Food Safety Area of the Ministry of Economy of the Republic of Armenia (March 15, 2022; Protocol No. 3). The animal sera utilised in this study were collected in 2020 as part of a national Armenian surveillance programme. Ethical approval for the national surveillance programme was granted by the Veterinary Department of the FSIB, the Republican Center for Veterinary‐Sanitary and Phytosanitary Laboratory Services, and the Veterinary Department of the Ministry of Economy (MoE) of Armenia. The field animal sampling and survey were undertaken by the local and central veterinarians of the Center for Agricultural Services under the MoE. Veterinary legislation in Armenia enables the departments of MoE and FSIB to perform research that supports the control and elimination of contagious animal disease. The study is reported in accordance with ARRIVE guidelines. Written consent for participation was obtained from the animal owners from the various regions of Armenia.

## Conflicts of Interest

The authors declare no conflict of interest.

## Data Availability

The data that support the findings of this study are available from the corresponding author upon reasonable request.
